# The Relationship Between Knee Pain and Heart Failure in Older Black and Latino Women

**DOI:** 10.1093/geroni/igab046.641

**Published:** 2021-12-17

**Authors:** Raya Kheirbek, Bernadette Siaton, Brock Beamer, Jacob Blumenthal, Les Katzel, John Sorkin, Beth Hogans

**Affiliations:** 1 University of Maryland School of Medicine, University of Maryland School of Medicine, Maryland, United States; 2 University of Maryland School of Medicine, Baltimore, Maryland, United States; 3 Baltimore Veterans Affairs Medical Center, BALTIMORE, Maryland, United States; 4 University of Maryland, Baltimore VA Medical Center, Maryland, United States; 5 Johns Hopkins University, Johns Hopkins University, Maryland, United States

## Abstract

Background: Knee pain is the second-most prevalent and disabling common pain condition globally, having deleterious effects on daily function including mobility and exercise capacity; chronic knee pain is especially prevalent in older adults. There is substantial evidence to indicate that physically inactive individuals have higher rates of cardiovascular disease. Nonetheless, studies investigating cardiovascular risks with osteoarthritis have had mixed results. Objective: This study explores the relationship between knee pain and heart failure especially examining the factors of age, gender, race in U.S. older adults. Methods: Retrospective secondary analysis of Medicare claims data for 1.478 million adults over age 65. The standard analytical file for 2017 was segmented according to the presence of any of several ICD-10 codes for heart failure (HF). Medicare beneficiaries with and without HF diagnoses were evaluated for knee pain and other common pain-associated conditions; pain condition data was stratified by age, gender and race codes. Results: Knee pain was markedly increased in women with HF in the 65-70- and 70–75-year-old age-cohorts and relatively less increased in older age-cohorts and males. Knee pain in women was especially elevated in those with Medicare race codes indicating Black and Hispanic status. Conclusion: in a large cohort of Medicare beneficiaries, knee pain was noted to be markedly increased in younger cohorts of older women with HF, and more prevalent in Black and Hispanic women. Further studies should evaluate lifestyle, biomechanics, and inflammatory factors that may be contributing to this relationship.

